# Dimensional Changes in the Alveolus after a Combination of Immediate Postextraction Implant and Connective Grafting and/or Socket Shield Technique

**DOI:** 10.3390/ijerph19052795

**Published:** 2022-02-27

**Authors:** Ramón Gómez-Meda, María Rizo-Gorrita, María-Angeles Serrera-Figallo, Jonathan Esquivel, Cristina Herraez-Galindo, Daniel Torres-Lagares

**Affiliations:** 1 Stomatology Department, Faculty of Dentistry, Sevilla University, C/Avicena s/n, 41092 Seville, Spain; ramon.gomez.meda@gmail.com (R.G.-M.); mrizog@us.es (M.R.-G.); crisnach.15@gmail.com (C.H.-G.); 2 Department of Prosthodontics, Louisiana State University School of Dentistry, New Orleans, LA 70119, USA; jesqu1@lsuhsc.edu

**Keywords:** immediate implant placement, connective tissue graft, socket shield technique, alveolar bone dimensional changes

## Abstract

Immediate implant placement protocols after dental extraction have enabled a reduction in surgical phases. This procedure has increased patient satisfaction and similar survival rates to late implant placement procedures. However, placing an implant immediately after dental extraction does not counteract the physiological remodeling of alveolar bone. For this reason, additional surgical techniques have been developed, such as the placement of a connective tissue graft (CTG) or the socket shield technique (SST). Dimensional changes in the peri-implant tissues were observed after placement of immediate implants following the extraction and CTG and/or SST. A total of 26 surgical interventions were carried out in which dimensional change variables of peri-implant tissues were analyzed. The preoperative state and immediate postoperative situation were compared with the situation after one year. Measurements were taken at 3, 5, and 7 mm from the gingival margin and analyzed in this CBCT radiological study (Planmeca Promax 3D). The implant platform was used as a reference point for the measurement of changes in alveolar crest height. One year after performing either of the two techniques (CTG and/or SST), a significant increase in the gingiva thickness and vestibular cortex occurred at 5 mm (0.65 ± 1.16 mm) and 7 mm (0.95 ± 1.45 mm) from the gingival margin. Additionally, an increase in thickness of palatal bone was registered at 3 mm (0.48 ± 0.90 mm). The graft placement group showed an increase in thickness of peri-implant tissue in the vestibular area after one year, although CTG and SST groups were clinically similar. The implementation of SST revealed promising results regarding the buccal thickness of hard and soft tissues after one year. A significant increase in vestibular cortical bone thickness, as well as the overall mucosa thickness and buccal bone at 3 mm from the gingival margin, was observed. A significant reduction in the distance from the bone crest to the platform was detected in both techniques. Both techniques (CTG and SST) are appropriate to provide sufficient volume to peri-implant tissues in the vestibular area of anterior maxillary implants. Some limitations were detected, such as the lack of an aesthetic analysis or small sample size, so results should be interpreted with caution. Future studies are necessary to further evaluate the long-term predictability of these techniques.

## 1. Introduction

The importance that implantology has acquired in recent years has boosted the emergence of minimalist and rapid techniques. Extraction of teeth with poor prognosis, followed by a simultaneous implant placement, saves time and costs, in addition to reducing morbidity in patients. However, at the same time, patients demand highly aesthetic results that blend in with their surrounding teeth and soft tissues. Immediate placement after dental extraction has reduced surgical times and increased patient satisfaction, with similar survival rates to those cases of late implant placement [1,2]. It is important to place an immediate implant in type I/II alveolus, with sufficient vestibular and palatine thickness of 1–2 mm to accommodate the implant and avoid mucosal fenestration and bone recession. It is also important that there is a bone in the apical and interproximal area of the alveolus [3]. Implant placement must be prosthodontically driven to avoid esthetic complications [4]. Interim restoration has to be also properly shaped to allow supracrestal tissues’ attachment to be reestablished at the time soft tissues are contoured [5].

Periodontal ligament stem cells have essential functions in maintaining and remodeling the periodontal complex [6]. After extracting a tooth, there is progressive remodeling of the bundle bone and the internal part of the alveolar bone, which loses vascularization from the periodontal ligament. Consequently, buccal bone dimensions change mainly during the first three months after dental extraction [7,8].

Alternative and/or additional techniques often need to be applied to compensate for socket resorption after tooth extraction. Immediate implant placement does not counteract physiological remodeling of the alveolar bone and does not provide any advantage over the implant placement in healed sites, where bone remodeling has reshaped the bone crest mainly horizontally [9,10,11]. For this reason, additional techniques have been developed, including connective tissue graft between the facial bone table and the flap [2,6].

A recent meta-analysis study evaluated the effects of available surgical procedures for preserving peri-implant tissues after the placement of immediate implants. It was concluded that connective tissue grafts prevent marginal bone loss and improve aesthetics [12].

Another technique that minimizes buccal bone resorption is the socket shield technique. It was described by Huzeler et al. in 2010, and its aim is to preserve the irrigation from a periodontal ligament by maintaining a coronal portion of the root in the vestibular area. After performing a vertical dental section in the mesiodistal direction, the palatal portion is removed, the root volume is reduced afterward, and the immediate implant is placed into the socket. Implants can be placed in direct contact with the tooth shield, or a gap can be left for a blood clot or bone graft. The integrity of the bundle bone is preserved by keeping the periodontal ligament in this area [13]. The formation of new bone and cement between radicular dentine and the surface of the implant has been reported [14,15,16]. The technique does not interfere with implant osseointegration [14], nor does the placement of grafts into the gap between the implant and the partial root, which compensates for the loss of soft and hard tissues after tooth extraction [17].

Several publications have investigated the tridimensional changes after immediate implants placed with SST or CTG, but no study has compared both techniques on immediate implants [18,19,20]. Therefore, this study aimed to observe the dimensional changes in peri-implant tissues after the placement of immediate postextraction implants and the performance of connective tissue graft and/or socket shield.

## 2. Materials and Methods

In the present single-center, prospective cohort study, a total of 15 patients were selected following the inclusion and exclusion criteria. In this way, smokers, diabetics, patients with immunological alterations, or those taking bone-affecting drugs (e.g., bisphosphonates) were excluded from the study. Healthy patients and patients without medical conditions that could affect bone remodeling were included. 

All patients included in this study accepted to participate after signing informed consent. All interventions were explained to participants, and they were able to understand the postoperative cares they had to carry out. 

Patients presented one or more teeth in the maxilla’s anterior area, which had a poor prognosis and had to be extracted and replaced by immediate implants. Some of the patients received more than one implant. Therefore, a total of 26 surgical sites were registered.

**Figure 1:**
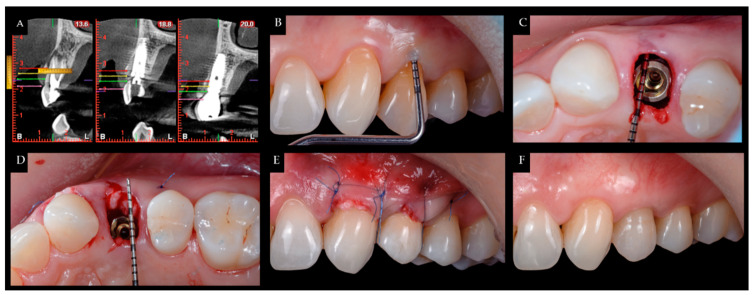
(**A**) CBCT measurement of the gingival margin (pink line), and marks at 3, 5, and 7 mm (green, yellow, and red lines, respectively); (**B**) crestal height location; (**C**) distance from the bone crest to the implant platform; (**D**) tunneling of a connective tissue graft; (**E**) suture; (**F**) one-year postoperative.

**Figure 2:**
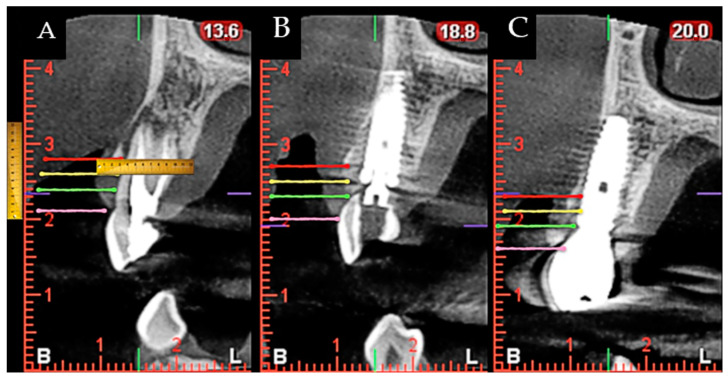
Image that identifies the locations of the measurements made in the CBCT during the study. CBCT measurement of the gingival margin (pink line), and marks at 3, 5, and 7 mm (green, yellow and red lines, respectively); crestal height location; distance from the bone crest to the implant platform; tunneling of a connective tissue graft; suture; one-year postoperative. (**A**) Preoperative situation; (**B**) Immediate postoperative situation; (**C**) One year follow-up.

During surgery and implant placement, a connective tissue graft was harvested, and/or a socket shield technique was carried out. Both techniques are described below. The bone crest dimensions were analyzed with a periodontal probe during surgery. Based on previous publications, the changes in peri-implant tissues volume were measured using a CBCT radiological study (Planmeca Promax 3D, Helsinki, Finland) immediately after implant placement and at check-ups by a single well-trained examiner (R Gómez-Meda) to ensure measurement consistency [21]. CBCT images were undertaken based on the radiological images of the Planmeca viewer software (Planmeca Romexis v. 3.8.3.R, Helsinki, Finland) (Figure 1 and Figure 2). The study was undertaken in Meda Dental Clinic (Ponferrada, Leon, Spain). 

### 2.1. Ethical Considerations

This study was conducted following the ethical principles for medical research on humans, as defined in the Declaration of Helsinki, and following the standards of good clinical practice. Approval was obtained from the Ethical Committee of Virgen del Rocío and Virgen Macarena Hospital (DTL-OXT-17).

### 2.2. Inclusion Criteria

The study included patients for whom the extraction of an upper front tooth with very poor prognosis. The patients selected presented enough height and width of bone volume to allow for the placement of implants with a minimum diameter of 3.75 mm and a minimum length of 13 mm.

The criteria used to allocate patients in the connective tissue grafting group were as follows: an infection affecting the radicular walls or suspicion of this and/or poor condition of the residual dentine of the tooth to be extracted.

The criteria used to consider the socket shield technique were as follows: dental root without a fracture, no presence of old posts or lateral canals, and no presence of infection affecting the buccal side of the root. 

### 2.3. Exclusion Criteria

Patients not included were those presenting any systemic pathology or health condition that would inhibit the osseointegration process of the implants, drug or alcohol abuse, poor oral hygiene, or lack of buccal wall in the extraction area (types III and IV of Elian’s classification) [22].

### 2.4. Variables Analyzed

The following features were measured at the preoperative stage, immediately after tooth extraction and one year after the intervention. Furthermore, the use or not of a connective tissue graft and/or socket shield was registered. All patients with previous periodontal disease were treated before implant placement, and clinical measurements were performed directly using a digital caliber or a periodontal probe (Table 1 and Table 2).

### 2.5. Connective Tissue Graft Technique

A 1 mm thick and 5 mm wide connective tissue graft was harvested from the palate after local anesthesia with 40 mg/mL + 0.01 mg/mL of articaine. The length of the CTG was equal to that of the site to reconstruct measured from the distal to the mesial papilla. The harvested CTG was tunneled into a pouch made with a microblade between the labial mucosa and the cortical plate of the socket. Two horizontal mattress sutures in the mesial and distal aspects of the sockets were used to stabilize the CTG in position. An immediate interim restoration or a customized healing cap was used to help stabilize the blood clot protecting and covering the CTG. In cases in which a minor gingival recession was detected, the tunneled flap was coronally displaced with sling sutures to fully cover the CTG and prevent its necrosis.

### 2.6. Socket Shield Surgical Technique

After local anesthetic in the area with 40 mg/mL + 0.01 mg/mL of articaine, a coronal section of the tooth was made using a Komet (Ref: H254314010) bur, and subsequently, another vertical division was made to section the vestibular and palatal portions of the root, removing the latter. The vestibular portion of the root was grounded down and chamfered with a diamond bur, Intensiv (Ref: FGD6/6C) to locate it at 3 mm from the gingival margin. The implant bed was drilled in the alveolus palatal area, leaving a 1–2 mm gap between the root and the implant platform. When the gap between the implant body and the remaining root shield was larger than 1–2 mm due to anatomical variables, a collagen sponge was used to stabilize the blood clot, but no biomaterial was utilized. Patients were recalled regularly at two weeks, one month, three months, six months, and one year after the intervention, although only results from 1 year after surgeries period was analyzed.

### 2.7. Implant Placement

The following internal hexagon bone level implants were purchased: Tri-vent^®^ SBA surface, BioHorizons^®^ (Birmingham, AB, USA) Tapered Internal Plus Laser-Lok surface, and Mozo Grau^®^ Inhex STD with RBM surface (Valladolid, Spain). The torque achieved at implant placement determined the immediate restoration used. When the torque was higher than 40 Ncm (measured with the handpiece), an immediate interim restoration was used to load the implant, but a customized healing cap was used with lower torques. 

### 2.8. Restorative Protocol

Three months after the implant placement, an impression was made to fabricate the final restoration, and two weeks later, the implant was finally restored. Zirconia crowns using the original manufacturer interface were made. Pre- and postoperative conditions were measured using a periodontal probe, CBTC, and visual exploration. Outcomes were analyzed by experienced surgeons and compared with a one-year follow-up. 

### 2.9. Statistical Analysis

The statistical significance of the results obtained was calculated using IBM SPSS Statistics 24.0 (International Business Machines Corp; New York, NY, USA). A complete descriptive analysis of the previously mentioned features was carried out. The Shapiro–Wilk test was performed to check the variables. Student’s *t*-test was used to compare the normal distribution of the variables and the Signos test for comparisons in which one of the variables did not show normal distribution. 

To analyze the existing differences between qualitative variables, a chi-squared test was applied.

Variance homogeneity was ascertained using Levene’s test. The ANOVA variance test was used to compare means across groups, and the Mann–Whitney U test was used for variables that were not consistent with normal distribution.

A 5% (*p* < 0.05) statistical significance level was established. 

The sample size was estimated based on other similar articles and the difficulty of finding patients who met the inclusion and exclusion criteria. It was decided to include a total of 15 patients after more than a year dedicated to the inclusion of patients. 

## 3. Results

### 3.1. Patient Determinants

Results relating to patient characteristics and type of surgery carried out are listed in Table 3. In total, 15 patients were treated between January 2019 and April 2020, but a total of 26 implants were placed. None of the 15 selected patients dropped out of the study. 

The mean age of the patients was 57.80 ± 11.77 years. Five patients were smokers consuming an average of 4.20 ± 1.64 cigarettes a day.

### 3.2. Implant Bed Variables

A total of 26 surgical sites were studied, considering that the average number of surgical interventions per patient was 1.08 ± 0.27 and that more than one implant was placed in some of the patients. Table 4, Table 5 and Table 6 analyze the variables after implant placement at the preoperative stage and the differences found after one year. Variables were measured at 3, 5, and 7 mm from the gingival margin using a periodontal probe to obtain mucosa thickness. 

### 3.3. Patient-Related Variables Based on Existence of a Subepithelial Connective Tissue Graft

Table 7 shows the percentages of the medical history and characteristics of the surgery variables in those patients who underwent graft surgery.

The mean age of patients who underwent a CTG was 56.80 ± 9.30 years. The mean age for those who were not subjected to a CTG was 56.20 ± 16.90 years.

### 3.4. Patient-Related Variables Based on Applying a CTG

The mean number of surgical interventions per patient was 1.10 ± 0.30 (with graft) and 1 (no graft). 

Table 8, Table 9 and Table 10 show the results obtained with respect to the performance of the technique on the variables analyzed for implants at the preoperative stage, as well as the differences encountered after one year.

### 3.5. General Patient-Related Variables, Based on Performance of Socket Shield Technique

Table 11 shows the results in those patients who were intervened with socket shield techniques.

The mean age of patients who underwent the socket shield technique was 58.25 ± 6.95 years. 

The mean age for those who were not subjected to the socket shield technique was 57.64 ± 13.40 years.

### 3.6. Implant-Related Variables in Those Sites with Socket Shield

The mean number of surgical interventions on patients with an SST was 1 and 1.10 ± 0.31 for those patients without SST. 

Table 12, Table 13 and Table 14 show the implant-related variables and the results obtained in patients with SST at the preoperative stage, as well as the differences found after one year.

No implant failures or complications, such as graft necrosis or mobility of the buccal shield, were registered during extraction of the root, implant placement, or during the postoperative period.

## 4. Discussion

The lack of hard and soft tissues around the implant at the time of its placement leads to the formation of aesthetic defects, such as the appearance of grey shadows [17,23,24] or gingival recessions [25,26]. The development of new techniques that enable peri-implant tissues to be maintained has shown to be a great advance. CTG and SST techniques have the advantage of maintaining buccal volume, promoting a better aesthetic result. On the other hand, an experienced operator is needed to carry out this type of surgery, especially SST. To date, there is limited evidence on the combination of socket shield technique (SST) and a connective tissue graft (CTG) [27,28]. These two techniques are usually compared with each other for the management of dimensional changes in peri-implant tissues [28,29]. 

This clinical study evaluated dimensional changes in peri-implant tissues when CTG and/or SST were performed at the time of immediate implant placement. To the authors’ knowledge, no comparative clinical study or combination of both techniques has been published to evaluate dimensional changes using CBCT and follow-up after one year. 

A total of 26 surgical interventions were carried out in which patient-dependent variables were analyzed after the implant placement. The preoperative state was compared with the immediate postoperative condition, and the outcome after year, both overall and compared with taking the graft and/or SST. Since the thickness of the peri-implant tissues varies throughout the vertical axis [30,31], measurements were taken at 3, 5, and 7 mm from the gingival margin. The implant platform was used as a reference point measuring changes in alveolar crest height.

After one year, it was observed that an increase in the thickness of mucosa and buccal cortex occurred at 5 and 7 mm from the gingival margin. An increase in thickness of palatal bone was registered at 3 mm (Table 4). This could be due to better horizontal maintenance in the buccal area by either the graft or the SST [12,17,32,33].

From the results observed in this study, the performance of CTG and SST as complementary techniques to the placement of immediate implants is sufficient for gaining and/or maintaining the volume of peri-implant tissue in the vestibular area of the implants. Despite the study’s limitations, such as the small sample size or lack of aesthetic analysis of the cases, this study may serve as a starting point for future studies to evaluate the predictability of this type of technique in the long term.

The literature has widely reported the efficiency of connective tissue graft placement to improve peri-implant aesthetics [34,35,36]. It is the treatment of choice for increasing the volume of soft tissues [36,37]. The palate was chosen as the donor area, due to its high predictability, scientific evidence, and good aesthetic results [38]. 

Yoshino et al. and Migliorati et al. compared immediate implants with and without a subepithelial CTG and observed that significantly lower marginal bone loss was present in those for which a CTG was used. Additionally, the mean facial gingival change was less pronounced in the group mentioned earlier [31,39].

In the present study, the grafted group showed increased buccal peri-implant tissue thickness after one year, although CTG and SST groups were clinically similar. These results coincide with those published for a recent meta-analysis study in which it was concluded that CTG contributes to buccal soft tissue stability and should be considered in the case of the risk of recession, such as patients with a thin gingival biotype of <0.5 mm buccal bone thickness [40]. Furthermore, a significant reduction was observed in palatal mucosa thickness, and the overall measurement of palatal mucosa and bone was measured at 3 mm from the gingival margin (Table 8), indicating losses of 0.49 and 0.04 mm, respectively. These reductions in thickness were also observed in the measurements at 5 and 7 mm (Table 8). In addition, a reduction was also observed in the mucosa and palatal cortex thickness in both intra-groups with SST or not (Table 12). The explanation for this finding may be attributed to the lack of tissue preservation techniques in that area, which are usually carried out in the buccal area of the implants, the cortical one being the most affected [40]. According to the literature reviewed, after immediate implant placement, the expected horizontal resorption of the palatal cortical layer after one year is approximately 0.62 mm [41]. 

Alveolar resorption occurs more markedly in a horizontal dimension, followed by vertical changes in medial buccal and medial lingual areas. Buccal cortical thickness is related to the degree of resorption after tooth extraction, the greater thickness being a protective factor for bone remodeling [30,40,42,43]. 

According to a recent meta-analysis study, the mean buccal cortical thickness in maxillary incisors and canine teeth is ≤1 mm, and in maxillary premolars, 1–2 mm [30]. Our results are in line with this finding (Table 4). No significant differences were observed at the preoperative stage in either buccal or palatal cortical thickness concerning subsequent placement or not of a graft (Table 8). Furthermore, only a statistically significant difference at the preoperative stage was observed in buccal bone thickness from the measurement at 5 mm, compared with the subsequent performance of the SST (Table 8: 1.87 ± 1.71 mm SST vs. 0.81 ± 0.87 mm with no SST), and this did not translate after one year into a difference between both groups (Table 8). As a result, both techniques were satisfactory for maintaining a similar level of vestibular and palatal cortical thickness between the two comparison groups.

According to the meta-analysis carried out by Lee et al., bone dimensions around the immediate implant show a reduction of approximately 0.5–1.0 mm on the vertical and horizontal facets, at 4–12 months after surgery [41]. After one year, a significant reduction between 0.31 and 0.84 mm was noted in the distance from the bone crest to the implant platform, not only at buccal, palatal, and distal aspects. These changes occurred irrespective of the technique (CTG or SST) used (Table 5). Buccal cortical height affects the position of the peri-implant gingival margin [41,44]; this was observed in the present study, together with a significant increase in the distance to the zenith of the contralateral tooth, with a mean gingival recession of 0.26 mm (Table 5). These dimensional changes are expected after placing an immediate implant, irrespective of whether tissue preservation techniques are performed. In a prospective study on 30 patients with a follow-up at 2–5 years, it was observed that the expected retraction of the medial area of the gingival margin in immediate implants was 0.29 mm [45]. These values are in line with those obtained in the present study. The results of this study agree with a recent meta-analysis study in which significant differences were not found between immediate implants with or without CTG with regard to marginal bone resorption (*p* = 0.42) [20].

SST is relatively recent, and the current literature mainly presents articles about the performance of the technique and its modifications but little evidence of its long-term predictability [32,46,47,48,49].

Several publications reviewed dimensional changes in tissues by comparing SST with a control group of an immediate implant with or without a graft [50,51,52]. Other articles examined this technique and assessed the dimensional changes occurring with CBCT, but they are reports of one clinical case [27,53,54]. 

It is known that SST favors lower horizontal and vertical bone resorption when comparing it with an immediate implant with bone graft placement [33,51,52]. Tiwari et al. reported a reduction in the buccal bone of 0.03 mm after one year from performing SST, compared with a loss of 0.187 mm on immediate implant placement only, although both differences were not significant [51]. 

The horizontal and vertical bone resorption after immediate implant placement is around 0.5–1 mm [41].

In the present study, by performing SST with and without CTG, lower levels of resorption to those mentioned above were expected after one year. A significant increase was observed in buccal cortical bone thickness and the joint measurement of mucosa and buccal bone at 3 mm from the gingival margin, compared with those observed when not performing this technique (1.62–1.91 mm vs. 0.13–0.20 mm) (Table 12). It is worth mentioning that an increase in tissue thickness was observed in both groups on the buccal side (Yes: socket shield performed or No: connective tissue graft), with no statistically significant differences between either group. In addition, the distance from the crest to the implant platform in the mesial area increased significantly. However, this finding is deemed anecdotal by the authors since the expected reduction in crest height occurred one year after placement of the immediate implant (between −0.19 and −0.82 mm), irrespective of the type of technique employed (SST or CTG; *p* > 0.05). Nevertheless, a comparison of our results with those published in the literature is complicated due to the lack of published data and the existing disparity regarding methodology and results depending on with which other technique SST is compared [48,49]. According to some authors, SST has similar efficiency to immediate implants without a graft in the prevention of horizontal and vertical bone resorption [32]. Furthermore, Bramanti et al. compared height resorption after three 3 years of follow-up in SST and in immediate implants with bone graft and observed a statistically significant difference between both groups (0.6 mm vs. 1.11 mm, respectively; *p* < 0.01) [52]. Two publications compared SST with immediate implants without bone graft and observed lower horizontal and vertical bone loss in the SST group (*p* < 0.05) [17,34].

Possible complications of this technique are the need for removal of the radicular shield, its external exposure (due to mobility of the shield towards the restoration), or internal exposure (due to mobility of the shield toward the oral cavity) with the possibility of infection and bone loss [28]. In the present study, no implant failures or complications were observed, but this technique is operator-dependent and should be performed by expert surgeons [28,32].

It was decided to include a total of 15 patients after more than a year dedicated to the inclusion of patients. Although being a small number, the sample size of 15 patients is even larger than that in other studies [13,17,23]. In terms of the limitations of this study, its small patient sample can be highlighted. Nevertheless, statistically significant differences were observed. A way of improving the study would be to increase the number of patients or surgical interventions performed to obtain better results. Another limitation was the lack of randomization, as the cases were analyzed before deciding which technique to perform. Furthermore, the presence of a control group for each of the techniques performed would have given information about the bone behavior in a conventionally healed alveolus, in addition to providing additional study variables. Finally, it would be beneficial to carry out an aesthetic evaluation of the cases undertaken.

## 5. Conclusions

The results observed in this study indicate that the performance of CTG and SST as complementary techniques in the placement of immediate implants is sufficient for gaining and/or maintaining the volume of peri-implant tissue in the buccal area of the implants. Despite this study’s limitations, such as its small sample size or the lack of aesthetic analysis of the cases, it may serve as a starting point for future studies to evaluate the long-term predictability of this kind of technique.

## Figures and Tables

**Table 1:** General variables relating to the medical history.

Age		Number of Cigarettes/Day	
Existence of systemic disease in the medical history		Drugs taken	
Smoking	Prior periodontal disease		Socket shield technique performed
Performance of second surgical intervention			

**Table 2:** Variables relating to the implant location.

Distance to the Zenith of the Contralateral Tooth	Vertical Distance from the Bone Crest to the Platform of the Implant after Placement
Number of surgical interventions	
Measurement of the thickness of the mucosa and the bone cortex in vestibular and palatal areas, individually and jointly, at 3 mm, 5 mm, and 7 mm from the gingival margin.	
Thickness of the graft from connective tissue	
Alveolar bone width at crestal bone level	

**Table 3:** Patients’ general characteristics and presence of second surgical intervention.

Variables	Categories	Frequency	Percentage
Sex	Female	10	66.7
	Male	5	33.3
Age (classified)	Up to 50 years	6	40.0
	Over 50 years	9	60.0
General disease (not affecting ridge remodeling)	Yes	12	80.0
	No	3	20.0
Drugs Taken	Yes	11	73.3
	No	4	26.7
Smoking	Yes	5	33.3
	No	10	66.7
Existence of prior periodontal disease	Yes	6	40.0
	No	9	60.0
Socket shield technique	Yes	6	23.1
	No	20	76.9
Existence of second surgical intervention	Yes	4	15.4
	No	22	84.6

**Table 4:** Preoperative situation and one-year follow-up data. Mucosa thickness and cortex bone thickness were measured. Graft thickness and alveolar width were registered (P: palatal and lingual; V: facial and buccal).

Location	Variables	No.	Mean	SD
Preoperative at 3 mm from the gingival margin	Mucosa thickness V	26	1.49	0.73
	Bone cortex thickness by V	26	0.72	0.96
	Mucosa thickness + bone by V	26	2.21	1.24
	Mucosa thickness P	26	3.05	1.53
	Bone by P	26	0.71	0.78
	Mucosa thickness + bone by P	26	3.77	1.37
One-year follow-up at 3 mm from the gingival margin	Mucosa thickness V	26	0.12	0.68
	Bone cortex thickness by V	26	0.48	1.34
	Mucosa thickness + bone by V	26	0.60	1.63
	Mucosa thickness P	26	−0.17	1.54
	Bone by P	26	0.48 *	0.90
	Mucosa thickness + bone by P	26	0.31	1.53
Preoperative at 5 mm from the gingival margin	Mucosa thickness V	26	1.43	0.76
	Bone cortex thickness by V	26	1.05	1.17
	Mucosa thickness + bone by V	26	2.48	1.19
	Mucosa thickness P	26	3.22	1.35
	Bone by P	26	1.81	0.73
	Mucosa thickness + bone by P	26	5.04	1.56
One-year follow-up at 5 mm from the gingival margin	Mucosa thickness V	26	0.06	0.81
	Bone cortex thickness by V	26	0.59 *	0.95
	Mucosa thickness + bone by V	26	0.65 *	1.16
	Mucosa thickness P	26	−0.43 *	1.18
	Bone by P	26	0.13	0.95
	Mucosa thickness + bone by P	26	−0.30	1.70
Preoperative at 7 mm from the gingival margin	Mucosa thickness V	26	1.47	0.74
	Bone cortex thickness by V	26	1.21	1.01
	Mucosa thickness + bone by V	26	2.67	1.14
	Mucosa thickness P	26	3.30	1.45
	Bone by P	26	2.44	0.68
	Mucosa thickness + bone by P	26	5.74	1.65
One-year follow-up at 7 mm from the gingival margin	Mucosa thickness V	26	0.35	1.06
	Bone cortex thickness by V	26	0.60 *	1.01
	Mucosa thickness + bone by V	26	0.95 *	1.45
	Mucosa thickness P	26	−0.22	1.11
	Bone by P	26	0.03	0.84
	Mucosa thickness + bone by P	26	−0.19	1.61
Clinical data	Graft thickness	20	2.69	1.24
	Alveolar bone width—periodontal probe (crest)	26	9.81	3.27

* There is a significant difference between the original variables.

**Table 5:** Preoperative and one-year follow-up data. Distance to the zenith of the contralateral tooth.

Distance to the Zenith of the Contralateral Tooth	No.	Mean	SD
Preoperative	13	8.07	1.80
Between 1 year and preoperative	13	0.26 *	1.24

* There is a significant difference between the original variables.

**Table 6:** Vertical distance from the bone crest to the implant platform.

Moment	Variables	No.	Mean	SD
Immediate	Mesial	26	2.38	1.31
	Distal	26	2.29	1.46
	Palatal	26	3.30	2.08
	Buccal	26	3.08	1.69
Between 1 year and preoperative	Mesial	26	−0.39	1.01
	Distal	26	−0.31 *	0.50
	Palatal	26	−0.87 *	1.57
	Buccal	26	−0.84 *	1.68

* There is a significant difference between the original variables.

**Table 7:** General variables relating to the patient (qualitative), based on the existence of graft.

Variable	Categories	Yes		No	
		Freq.	Perc.	Freq.	Perc.
Existence of systemic disease	Yes	13	61.9	5	100.0
	No	8	38.1	0	0.0
Drugs Taken	Yes	13	61.9	4	80.0
	No	8	38.1	1	20.0
Smoking	Yes	4	19.0	3	60.0
	No	17	81.0	2	40.0
Existence of prior periodontal disease	Yes	9	42.9	2	40.0
	No	12	57.1	3	60.0
Socket shield	Yes	5	23.8	1	20.0
	No	16	76.2	4	80.0
Existence of second surgical intervention	Yes	4	19.0	0	0.0
	No	17	81.0	5	100.0

**Table 8:** Preoperative and one-year follow-up data based on graft placement or not. Mucosa and cortical bone thickness at 3, 5, and 7 mm from the gingival margin. Graft thickness and alveolar width were registered. (P: palatal and lingual; V: facial and buccal). (Sign., Significance).

Location	Variables	Yes		No		Sign.
		Mean	SD	Mean	SD	
At 3 mm from the gingival margin	Mucosa thickness V	1.51	0.71	1.42	0.89	
	Bone cortex thickness by V	0.60	0.92	1.22	1.10	
	Mucosa thickness + bone by V	2.11	1.10	2.64	1.79	
	Mucosa thickness P	3.33	1.54	1.90	0.82	
	Bone by P	0.73	0.83	0.64	0.59	
	Mucosa thickness + bone by P	4.06	1.34	2.54	0.59	<0.05
One-year follow-up at 3 mm from the gingival margin	Mucosa thickness V	0.22	0.66	−0.32	0.66	
	Bone cortex thickness by V	0.37	1.31	0.92	1.52	
	Mucosa thickness + bone by V	0.59	1.70	0.61	1.49	
	Mucosa thickness P	−0.49	1.45	1.17	1.23	<0.05
	Bone by P	0.45	0.97	0.64	0.53	
	Mucosa thickness + bone by P	−0.04	1.39	1.80	1.25	<0.05
At 5 mm from the gingival margin	Mucosa thickness V	1.47	0.77	1.25	0.75	
	Bone cortex thickness by V	0.95	1.20	1.50	1.00	
	Mucosa thickness + bone by V	2.42	1.11	2.75	1.64	
	Mucosa thickness P	3.36	1.32	2.64	1.47	
	Bone by P	1.93	0.70	1.30	0.67	
	Mucosa thickness + bone by P	5.30	1.53	3.94	1.30	
One-year follow-up at 5 mm from the gingival margin	Mucosa thickness V	0.09	0.88	−0.05	0.51	
	Bone cortex thickness by V	0.53	0.98	0.83	0.87	
	Mucosa thickness + bone by V	0.62	1.18	0.78	1.20	
	Mucosa thickness P	−0.62	1.10	0.40	1.27	
	Bone by P	0.06	1.03	0.44	0.44	
	Mucosa thickness + bone by P	−0.57	1.65	0.84	1.54	
At 7 mm from the gingival margin	Mucosa thickness V	1.50	0.77	1.34	0.65	
	Bone cortex thickness by V	1.10	0.93	1.68	1.32	
	Mucosa thickness + bone by V	2.59	0.98	3.02	1.78	
	Mucosa thickness P	3.37	1.39	3.00	1.84	
	Bone by P	2.51	0.63	2.15	0.87	
	Mucosa thickness + bone by P	5.88	1.61	5.15	1.91	
One-year follow-up at 7 mm from the gingival margin	Mucosa thickness V	0.39	1.16	0.18	0.47	
	Bone cortex thickness by V	0.51	1.00	0.96	1.09	
	Mucosa thickness + bone by V	0.90	1.56	1.14	0.98	
	Mucosa thickness P	−0.35	1.15	0.30	0.84	
	Bone by P	0.02	0.88	0.09	0.70	
	Mucosa thickness + bone by P	−0.33	1.67	0.38	1.27	
Clinical data	Graft thickness	---	---	---	---	
	Alveolar bone width—periodontal probe (crest)	10.32	3.27	7.69	2.52	

**Table 9:** Preoperative and one-year data based on the presence of a graft. Distance to the zenith of the contralateral tooth (Sign., Significance).

Distance to the Zenith of the Contralateral Tooth	Yes		No		Sign.
	Mean	SD	Mean	SD	
Preoperative	8.05	2.02	8.11	1.44	
Between 1 year and preoperative	0.41	1.07	−0.08	1.69	0.41

**Table 10:** Preoperative situation based on presence of a graft. Vertical distance from the bone crest to the implant platform after implant placement. (Sign., Significance).

Location	Variables	Yes		No		Sign.
		Mean	SD	Mean	SD	
Immediate	Mesial	2.34	1.30	2.54	1.44	
	Distal	2.39	1.59	1.83	0.58	
	Palatal	3.64	2.11	1.87	1.26	
	Buccal	3.43	1.69	1.63	0.56	<0.05
Between situation at one year and preoperative	Mesial	−0.21	0.92	−1.15	1.10	
	Distal	−0.35	0.53	−0.16	0.41	
	Palatal	−0.96	1.71	−0.53	0.81	
	Buccal	−0.96	1.85	−0.37	0.32	

**Table 11:** General patient-related variables (qualitative), based on the presence of socket shield. (Sign., Significance).

Variable	Categories	Yes		No		Sign.
		Freq.	Perc.	Freq.	Perc.	
Existence of systemic disease	Yes	6	100.0	12	60.0	
	No	0	0.0	8	40.0	
Drugs Taken	Yes	6	100.0	11	55.0	<0.05
	No	0	0.0	9	45.0	
Smoking	Yes	2	33.3	5	25.0	
	No	4	66.7	15	75.0	
Existence of prior periodontal disease	Yes	2	33.3	9	45.0	
	No	4	66.7	11	55.0	
Existence of second surgical intervention	Yes	2	33.3	2	10.0	
	No	4	66.7	18	90.0	

**Table 12:** Preoperative and one-year follow-up data based on SST performance or not. Mucosa and cortical bone thickness at 3, 5, and 7 mm from the gingival margin. Graft thickness and alveolar width are noted. (P: palatal and lingual; V: facial and buccal). (Sign., Significance).

Location	Variables	Yes		No		Sign.
		Mean	SD	Mean	SD	
At 3 mm from the gingival margin	Mucosa thickness V	1.55	1.11	1.48	0.61	
	Bone cortex thickness by V	0.33	0.82	0.83	0.99	
	Mucosa thickness + bone by V	1.88	1.27	2.31	1.24	
	Mucosa thickness P	2.90	1.25	3.10	1.63	
	Bone by P	0.42	0.80	0.80	0.78	
	Mucosa thickness + bone by P	3.32	0.87	3.90	1.48	
One-year follow-up at 3 mm from the gingival margin	Mucosa thickness V	0.29	0.91	0.07	0.62	
	Bone cortex thickness by V	1.62	1.25	0.13	1.19	<0.05
	Mucosa thickness + bone by V	1.91	1.92	0.20	1.35	<0.05
	Mucosa thickness P	0.43	1.34	−0.35	1.57	
	Bone by P	0.32	0.37	0.53	1.00	
	Mucosa thickness + bone by P	0.76	1.60	0.18	1.52	
At 5 mm from the gingival margin	Mucosa thickness V	0.85	0.40	1.60	0.76	<0.05
	Bone cortex thickness by V	1.87	1.71	0.81	0.87	<0.05
	Mucosa thickness + bone by V	2.72	1.87	2.41	0.97	
	Mucosa thickness P	3.07	1.22	3.27	1.41	
	Bone by P	1.82	0.97	1.81	0.67	
	Mucosa thickness + bone by P	4.88	1.46	5.08	1.62	
One-year follow-up at 5 mm from the gingival margin	Mucosa thickness V	0.42	0.63	−0.04	0.84	
	Bone cortex thickness by V	0.78	1.21	0.53	0.89	
	Mucosa thickness + bone by V	1.19	1.29	0.49	1.10	
	Mucosa thickness P	−0.24	1.14	−0.48	1.22	
	Bone by P	−0.16	0.98	0.22	0.95	
	Mucosa thickness + bone by P	−0.40	2.03	−0.26	1.65	
At 7 mm from the gingival margin	Mucosa thickness V	0.85	0.29	1.65	0.74	<0.05
	Bone cortex thickness by V	1.80	1.08	1.03	0.95	
	Mucosa thickness + bone by V	2.65	1.20	2.68	1.16	
	Mucosa thickness P	3.02	1.19	3.38	1.54	
	Bone by P	2.32	0.63	2.48	0.70	
	Mucosa thickness + bone by P	5.34	1.21	5.86	1.77	
One-year follow-up at 7 mm from the gingival margin	Mucosa thickness V	0.68	0.84	0.25	1.12	
	Bone cortex thickness by V	0.70	1.07	0.57	1.02	
	Mucosa thickness + bone by V	1.38	1.50	0.82	1.45	
	Mucosa thickness P	0.07	1.14	−0.31	1.12	
	Bone by P	−0.24	0.90	0.11	0.83	
	Mucosa thickness + bone by P	−0.17	1.85	−0.20	1.58	
Clinical data	Graft thickness	2.60	0.55	2.72	1.40	
	Alveolar bone width—periodontal probe (crest)	8.02	4.34	10.35	2.78	

**Table 13:** Postoperative situation in sites with SST. Distance to the zenith of the contralateral tooth (Sign., Significance).

Distance to the Zenith of the Contralateral Tooth	Variables	Yes		No		Sign.
		Mean	SD	Mean	SD	
Preoperative		7.57	2.55	8.37	1.25	
Between 1 year and preoperative		0.65	1.36	0.01	1.18	

**Table 14:** Preoperative and one-year follow-up data in sites with SST. Vertical distance from the bone crest to the platform of the implant after placement. (Sign., Significance).

Location	Variables	Yes		No		Sign.
		Mean	SD	Mean	SD	
Immediate	Mesial	1.54	0.68	2.63	1.35	
	Distal	2.36	2.44	2.26	1.11	
	Palatal	2.24	1.81	3.61	2.09	
	Buccal	3.28	2.23	3.02	1.55	
Between situation at one year and preoperative	Mesial	0.38	1.51	−0.62	0.71	<0.05
	Distal	−0.19	0.38	−0.35	0.54	
	Palatal	−0.18	1.20	−1.08	1.64	
	Buccal	−0.82	2.94	−0.85	1.19	

## Data Availability

The data presented in this study are available on request from the corresponding author.

## Abbreviations

CTG: connective tissue graft; SST: socket shield technique; CBCT: cone-beam-computed tomography; RBM: resorbable blast media; SBA: surface sandblasted, large grit, acid-etched.
